# Distinct exercise modalities on GUT microbiome in sarcopenic older adults: study protocol of a pilot randomized controlled trial

**DOI:** 10.3389/fmed.2025.1504786

**Published:** 2025-03-05

**Authors:** Ana Sofia Merelim, Rodrigo Zacca, Daniel Moreira-Gonçalves, Paulo P. Costa, Liliana C. Baptista

**Affiliations:** ^1^Faculty of Sports, Research Center in Physical Activity, Health and Leisure (CIAFEL), University of Porto (FADEUP), Porto, Portugal; ^2^Laboratory for Integrative and Translational Research in Population Health (ITR), Porto, Portugal; ^3^Multidisciplinary Unit for Biomedicine Research (UMIB), University of Porto, Porto, Portugal; ^4^Department of Human Genetics, CSPGF, Instituto Nacional de Saúde Dr. Ricardo Jorge, Porto, Portugal; ^5^Faculty of Sports Sciences and Physical Education, University of Coimbra, Coimbra, Portugal; ^6^Research Center in Sport and Physical Activity (CIDAF), University of Coimbra, Coimbra, Portugal

**Keywords:** muscle quantity, muscle quality, aerobic exercise, resistance exercise, concurrent exercise, gut microbiota, sarcopenia, older adults

## Abstract

**Background:**

Sarcopenia is a progressive and age-related skeletal muscle disease related to adverse health outcomes and to an increased economic burden. Recent evidence pinpoints the human gut microbiota (GM) as a contributing factor in the development of sarcopenia via the gut-muscle axis. To date, no study specifically analyzed the optimal type of exercise modality in older adults with sarcopenia considering the impact of GM composition in skeletal muscle mass and function. Therefore, the DEMGUTS study intents to explore the impact of three different exercise regimens on GM composition and gut-derived metabolites in older adults with sarcopenia.

**Methods:**

This pilot single center three-arm parallel open-label randomized control trial (RCT) will randomly assign eligible participants to: (i) moderate aerobic exercise (AER); (ii) resistance exercise (RES); or (iii) concurrent exercise training (RES + AER). Participants will engage in a supervised center-based exercise intervention (12-weeks, 3 d/week, 60 min/d), and will be assessed at (i) baseline, (ii) end of intervention (14 weeks), and (iii) at close-out (26-weeks). The primary outcome will be the change in the relative abundance of *Faecalibacterium prausnitzii* and other short-chain fatty acid producing bacteria after the intervention (14-weeks). A set of complementary outcomes will also be assessed to broadly characterize the impact of each exercise intervention on body composition, skeletal muscle function, functional performance and general GM composition.

**Conclusion:**

Unraveling the impact of these exercise regimens on GM is crucial to help clarify the optimal exercise modality to manage sarcopenia disease, contributing to clinical guidance and enhancing exercise prescription in older adults with sarcopenia.

**Clinical trial registration:**

https://clinicaltrials.gov/, identifier NCT06545123.

## 1 Introduction

Sarcopenia is a progressive age-related skeletal muscle disorder (1–5) associated with adverse health outcomes, predictor of chronic disease progression, physical dysfunction, poor quality of life and premature death (4, 6–8). The prevalence of sarcopenia ranges from 10% in healthy adults over 60 years of age to 33% in people in long-term care (9). The mechanisms that contribute to the onset of sarcopenia are multifactorial and not fully understood (10). The combination of neurological factors – loss of motor neurons and motor units –, endocrine and lifestyle changes related to physical activity (PA) levels and nutrition also appear to contribute to the onset of sarcopenia (11). Hormonal and cytokine imbalances, age-related chronic inflammation (i.e., inflammaging) and metabolic disorders—namely insulin resistance have also been described as pathophysiological mechanisms involved in sarcopenia (11). The interplay of all these mechanisms reduces the expression of skeletal muscle anabolic factors and activate skeletal muscle catabolism pathways (11).

Recent evidence highlights the human gut microbiota (GM) as a new contributing mechanism to the development of sarcopenia (10). The decrease in gut biodiversity and function and the disruption in the composition of bacteria strains with health-promoting activity, such as *Bifidobacteria, Lactobacilli*, *Faecalibacterium prausnitzii, Eubacterium* spp., *Roseburia* spp., and *Ruminococcus* spp. are associated with the aging process and also to sarcopenia (12–15). Moreover, gut dysbiosis is associated to reduced muscle size, impaired muscle function and adverse clinical outcomes; the mechanisms underlying this relationship are still unclear.

It is known that gut microbiome composition may vary due to multiple endogenous and environmental factors such as diet, physical activity and medication or supplementation use and may confound the causal relationship between gut microbiome and sarcopenia (16). Still, gut dysbiosis contributes to gut barrier dysfunction, increasing its permeability, favoring the translocation of microbial byproducts such as lipopolysaccharides into the blood stream (1, 11, 17). This process aggravates age-related low-grade chronic inflammation and increases insulin resistance, mechanisms that are involved in the onset of sarcopenia (1, 11).

The effect of exercise on the GM composition has been analyzed qualitatively and quantitatively and is mostly associated with positive results in the aging process (18, 19). For instance, the increase in exercise levels is associated with a decrease in the relative abundance of potentially pathogenic taxa, higher microbiota biodiversity and butyrate production taxa (18), whereas a sedentary behavior is associated with gut dysbiosis, growth of pathogenic opportunists bacteria strains and alterations on GM functionality (20). Higher proportions of *Akkermansia, Faecalibacterium prausznitzii, Roseburia hominis*, and *Bifidobacterium spp*- known for its anti-inflammatory health benefits were observed in active women and athletes (14). Further, exercise training decreased the abundance of bacteria associated with intestinal dysbiosis, suggesting that exercise stimulates healthy gut microbes, in addition to improving muscle in physically active individuals (1). Despite these positive effects of exercise training on GM in young adults, the findings in older adults are less clear and conflicting, changes appear to be transient and reversible and to date, the effects of exercise training on GM in patients with sarcopenia remains unclear (21, 22).

The available evidence suggests that different types of exercise modalities may elicit distinct alterations in GM composition. Aerobic and resistance exercise activate different molecular pathways. Aerobic training (AER) improves the permeability of intestinal barrier, promotes bacterial taxa related to aerobic capacity and SCFA production whereas resistance exercise (RES) may enhance the composition of bacterial strains associated to muscle strength, upregulate anti-inflammatory cytokines and reduce endotoxemia (18, 23, 24). Given that these two exercise training regimens activate different bioenergetic signaling pathways [i.e., AER- activates the transient activated protein kinase (AMPK) pathway whereas RES activates the longer-lasting mechanistic target of rapamycin (mTOR)], we can hypothesize that each regimen promotes distinct alterations on GM, whereas the combination of both forms of exercise (resistance + aerobic) may lead to a complementary or added effect. There is limited evidence on the effects of concurrent training on sarcopenia outcomes, but the scarce evidence highlights the positive effects of combining both forms of exercise training on body composition, skeletal muscle regeneration and cardiorespiratory fitness (25). However, to date, no studies specifically analyzed the impact of different exercise training regimens (i.e., resistance, aerobic and concurrent exercise training) on GM composition and investigated its effects on skeletal muscle mass and function in older adults with sarcopenia. Thus, we designed a pilot three-arm randomized controlled trial (RCT) to unravel the effect of these exercise regimens on GM to help clarify the optimal exercise mode to manage sarcopenia and, we hypothesize that the combined exercise training mode may elicit more benefits on GM composition and function, on gut health and physical performance than each exercise training regimen alone.

## 2 Methods

### 2.1 Study design

The DEMGUTS project is a three-arm pilot RCT designed to evaluate the impact of different exercise training regimens on GM composition and on skeletal muscle mass and functionality in older adults with sarcopenia (age ≥ 60 years). This project was designed through a collaborative effort of a multidisciplinary team of experts in microbiome, exercise training and metabolomics. This project fulfilled all ethical procedures including the approval of the FADEUP ethics Committee (approval #CEFADE 4) and was registered on a public database (clinical trials: NCT06545123). This study followed strict guidelines to ensure participant safety and protection as recommended by the Helsinki Declaration (26).

### 2.2 Participant recruitment

Eligible volunteers will be recruited through an established collaboration with the community exercise program “*Mais e Melhores Anos*” at Famalicão. The inclusion criteria in our trial are based on the diagnosis criteria defined by the EWGSOP2 (27, 28) and thus, our goal is to enroll participants that have low skeletal muscle strength and quality. The assessment of eligibility will be made in two phases. In the first phase, prior to the screening visit, two members of the research team will analyze physical performance tests records collected in two different time points (October 2023 and June 2024), to identify eligible volunteers. Men and women who present handgrip test values below the sarcopenia cut-off point (men <27 kg and women <16 kg) will then be invited to an in-person screening visit to continue the eligibility assessment process. In this in-person assessment visit and prior to enrolment, − the second phase, each volunteer will receive a detailed explanation of all study phases and procedures, the benefits and risks along with all participant duties. Those that agreed to participate will provide their written informed consent as mandated by the Helsinki Declaration and its later amendments to protect human research (26). Afterwards, volunteers will be evaluated through additional complementary clinical and functional tests, including the assessment of body composition through dual-energy x ray absorptiometry, the Short Physical Performance Battery, the 4 meters gait speed, the 400 meters long distance corridor test, and through a handgrip dynamometry test—objective indicators of sarcopenia (27, 28), to confirm their eligibility. Those who met all the inclusion/exclusion criteria will be randomly assigned to one of three different exercise training interventions: (i) aerobic exercise (AER) training; (ii) resistance exercise (RES); or (iii) concurrent training (RES + AER).

#### 2.2.1 Inclusion/exclusion criteria

Participants will be included if: (*a*) aged ≥60 years; (*b*) present objective indicators of sarcopenia recommended by the EWGSOP2 [handgrip strength: men <27 kg and women <16 kg + appendicular skeletal muscle index/ height^2^: men <7.0 kg/m^2^ and women <5.5 kg/m^2^; severity will be assessed by the 4-m gait speed ≤0.8 m/s; SPPB ≤8-point score; Chair stand test >15 s; Time-up-go ≥20 s]; (*c*) level of PA ≤150 min/week of moderate PA assessed using the International Physical Activity Questionnaire (IPAQ)- Portuguese version (29); (*d*) Willingness to participate in all study procedures regardless of possible group allocation. In contrast, participants will be excluded if: (a) fail to provide consent; (b) present uncontrolled hypertension: (c) Systolic Blood Pressure (SBP) > 180 mmHg or Diastolic Blood Pressure (DBP) > 110 mmHg; (d) history of coronary artery stenosis (>50%); (e) heart failure: ejection fraction <50%; (f) history of syncope at exertion; (g) history of hypertrophic cardiomyopathy; arrhythmogenic cardiomyopathy, dilated cardiomyopathy or evidence in the previous 6 months of myocarditis or pericarditis; (h) severe valvular heart disease; (i) chronic kidney disease stage ≥4.8; (j) chronic obstructive pulmonary disease stage ≥3.9; (k) musculoskeletal or neurodegenerative conditions that hinders exercise engagement; (l) current participation in another structured exercise training program; (m) inability to commit to study procedures or the exercise intervention throughout the study period; (n) other condition or concern precluding safe participation.

#### 2.2.2 Sample size

The sample size was calculated based on a previous study (3) were authors saw a large effect after the intervention (Cohen d’s effect size = 1.15). Given that to date, there are no consensus in the optimal method to analyze microbiome health status, we selected the change on the relative abundance of *Faecalibacterium prausnitzii* and other SCFA (Short-Chain Fatty Acid) producing bacteria after the intervention (12-weeks) as our primary outcome. Nonetheless, this pilot study was powered to detect a more conservative effect size- of 0.9 on *Faecalibacterium prausnitzii* relative abundance after the intervention between experimental groups. Assuming an 80% power, a two-sided significance level of 0.05, we would need a total of 21 participants/group (calculated on GPower version 3.2). With an allocation ratio of 1:1:1 and an attrition rate of 10%, the final sample is of *N* = 69 (*n* = 23/group) participants.

#### 2.2.3 Randomization

Participants (total *N* = 69) will be randomized into three different groups. After the baseline assessment and eligibility confirmation, the participants study identification sheet will be sent to an independent study team member responsible for the randomization. This study member will generate a list of random numbers using Researcher Randomizer.^1^ This list will define entry order into the randomization process and will allow proper allocation sequence concealment and prevent selection bias. Eligible participants will be randomized to: (*i*) moderate aerobic exercise training [AER: 60–70% HRmax; n = 23]; (*ii*) resistance exercise [RES: 8 exercises/major muscle groups; 8–15 reps; 60–70% 1RM; *n* = 23]; and, (*iii*) a combination of both [RES + AER; *n* = 23] groups at baseline. The randomization will be with a 1:1:1 allocation ratio since all groups are active interventions. The selection of this study design with all “active” groups reduces the possibility of potentially inferior treatment/no treatment and increases volunteers’ acceptability to recruitment. After the randomization, the study team will inform the exercise interventionist about the ID allocation. Due to exercise specificities blinding of participants and exercise instructors is not feasible. However, group allocation will only be communicated to participants immediately before the intervention. The exercise instructors will not perform outcome assessments nor data analysis, and the outcome assessors will be blinded to allocation groups. Participants will be informed to not reveal randomization groups during assessment visits.

### 2.3 Intervention

**Figure 1:**
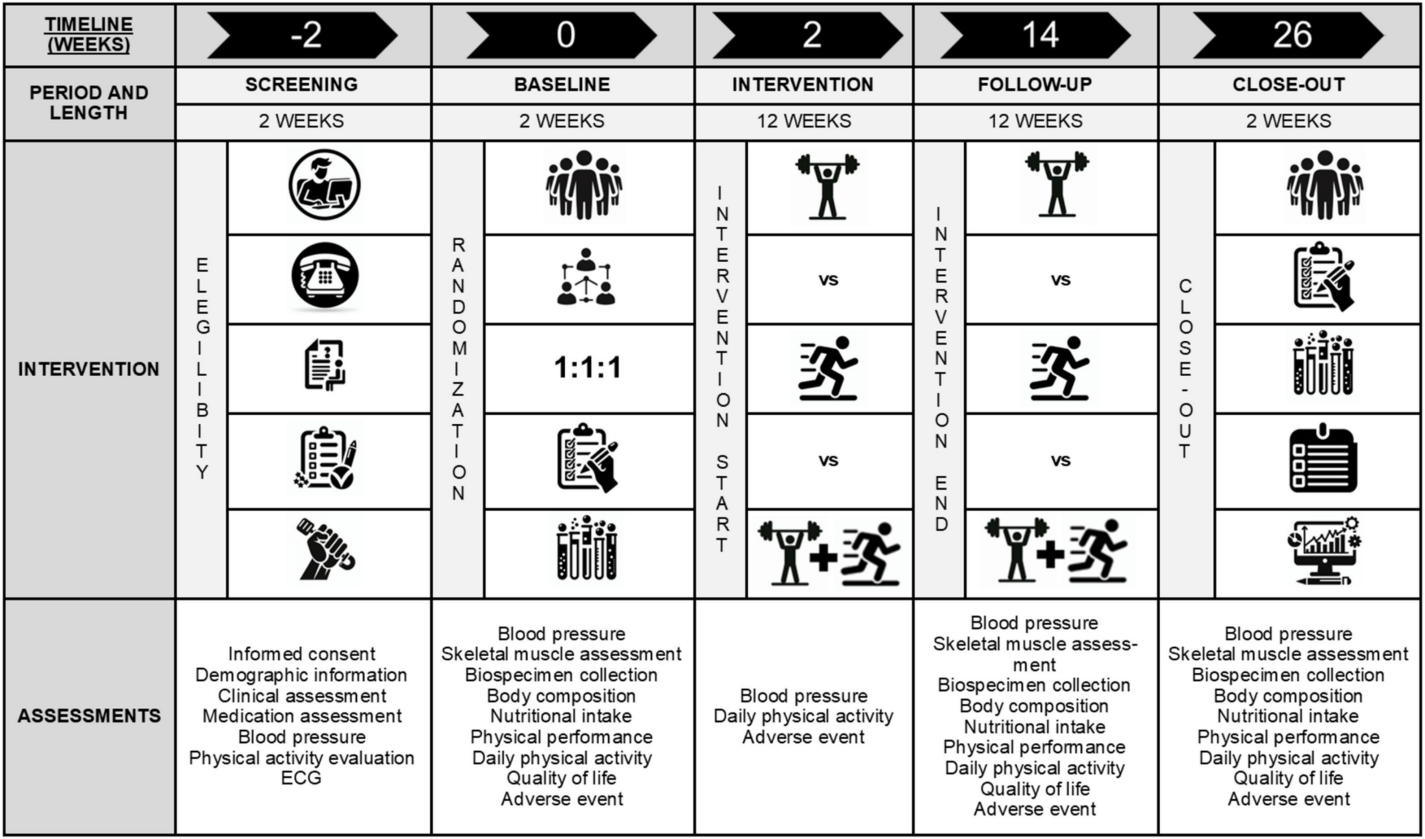
Descriptive scheme of the study flow and assessments.

The exercise intervention will be carried out in the city of Famalicão, 3 days/week, 60 min/day, on pavilions and at municipal gyms whereas the assessment procedures will take place at Faculty of Sport at the University of Porto, since the institution has specific facilities and equipment for this purpose. The intervention length is 12-weeks followed by one washout period of 12-weeks. The expected total study duration is ~8 months, including the baseline and the close-out procedures (Figure 1). The exercise sessions will take place in the morning, on alternating days of the week and will be supervised by experienced exercise physiologists. The sessions consist of a warm-up (5 min), a main part (50 min) and a cooldown (5 min). The warm-up and cooldown will be identical in all three groups, with only the main part being different between the groups.

**Figure 2:**
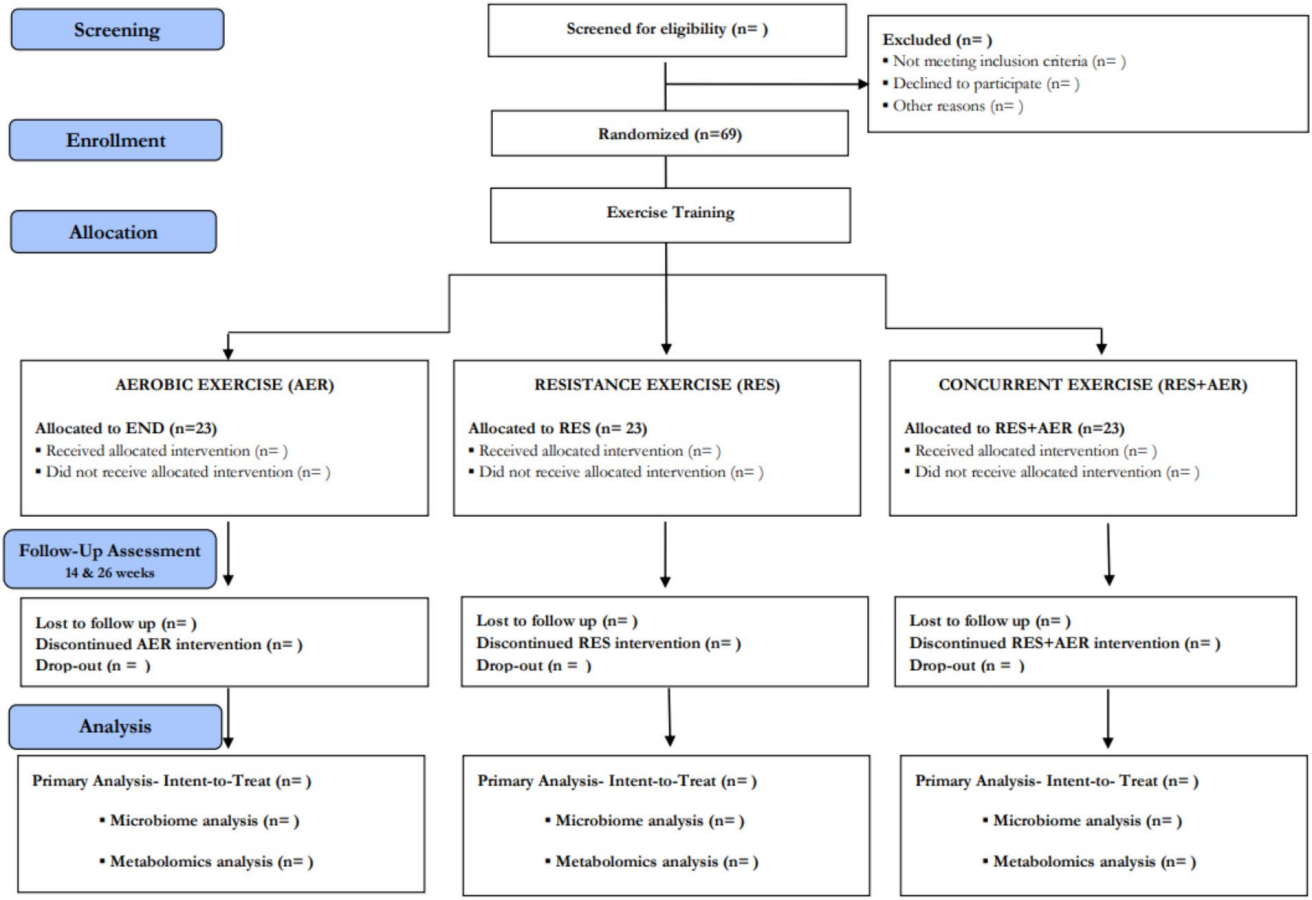
Consolidated Standards of Reporting Trials (CONSORT) diagram—overview of participants flow throughout the study.

Exercise intensity during the sessions will be measured using heart rate (HR) monitors and using the Borg Perceived Exertion Scale (30) across the different groups. The exercise interventions followed the *Consensus on Exercise Reporting Template* (CERT) guidelines (31). All instructors will undergo thorough training to standardize protocols prior to study beginning. All adverse events that occur during the exercise sessions will be recorded. When participants miss two consecutive sessions, they will be contacted by their instructor for support and evaluation. Motivational text messages will be sent to all participants to encourage their continued involvement. Although no other home exercise intervention will be provided to the experimental groups, daily home PA will be assessed in all groups using the FitBit^®^ Zip (San Francisco, CA, United States), and sedentary time, light, moderate and vigorous PA, and step count will be recorded—these data will serve as a covariate in the analysis of the primary outcome. Participants will be discontinued from the exercise intervention if significant adverse events occur [i.e., major cardiovascular (CV) events, hospitalization, major musculoskeletal injury, death or other life-threating situation] or if they engage in another structured exercise intervention (Figure 2).

#### 2.3.1 Resistance exercise protocol

For the RES group, the main part includes free weights, weight machines, and calisthenics; loads will be adjusted based on one repetition maximum (1RM) method. The exercises were standardized, however, when the participants presented limitations, these will be adapted according to participants cardiorespiratory fitness and functional abilities. The protocol for the RES intervention comprises an adaptation period of 3 weeks, followed by 9 weeks of the training-phase as recommended (2). Detailed characteristics of RES protocol regarding the muscle groups, intensity and loads are described in Table 1. The intensity in the RES group is going to be monitored by an HR monitor and by the Borg Perceived Exertion CR10 scale trying to attain an intensity between 5 and 7, which corresponds to a moderate exercise intensity.

**Table 1:** Exercise training plan and progression- RESISTANCE (RES) Group.

**Week**	**Session**	**Anatomic Region**		**N. Exercises**	**N. Sets**	**N. Reps**	**Load percentage (1RM)**
1	1	Upper limbs	Back	10	1	15	40
	2	Torso	Upper limbs				
	3	Core	Back				
2	4	Core	Torso	8	2	15–20	50
	5	Upper limbs	Back				
	6	Upper Limbs	Upper limbs				
3	7	Torso	Upper limbs	7	2	20	60
	8	Back	Core				
	9	Core	Torso				
4	10	Upper limbs	Upper Limbs	8	3	15	60
	11	Upper Limbs	Back				
	12	Torso	Upper limbs				
5	13	Back	Core	8	3	15	60
	14	Core	Torso				
	15	Upper limbs	Upper limbs				
6	16	Upper limbs	Back	8	3	10	70
	17	Torso	Upper limbs				
	18	Back	Core				
7	19	Core	Torso	8	3	10	70
	20	Upper limbs	Upper limbs				
	21	Upper limbs	Back				
8	22	Torso	Upper limbs	8	3	12	70
	23	Back	Core				
	24	Core	Torso				
9	25	Upper limbs	Upper limbs	8	3	12	70
	26	Upper limbs	Back				
	27	Torso	Upper limbs				
10	28	Back	Core	8	3–4	10–12	70
	29	Core	Torso				
	30	Upper limbs	Upper limbs				
11	31	Upper limbs	Back	8	3–4	12	70
	32	Torso	Upper limbs				
	33	Back	Core				
12	34	Core	Torso	8	3–4	12	70
	35	Upper limbs	Upper limbs				
	36	Upper limbs	Upper limbs				

#### 2.3.2 Aerobic exercise protocol

The AER exercise intervention will include walking/jogging in the treadmills, stationary and elliptical bikes in the main part of the sessions. Throughout the sessions, intensity will be measured by maximum HR (HRmax) and by the Rate of Perceived Exertion Scale (RPE 3–5) on category ratio (CR) 10 of Borg’s scale (30) progressing to RPE 6–7. The protocol for the AER intervention was divided into three period that are fully described in Table 2.

**Table 2:** Exercise training plan and progression-AEROBIC (AER) Group.

Week	Session	Device	N. Sets	Duration (min)	Rest between sets (min)	Load percentage (HRmax)
1	1	Treadmill	3	15	3	50
	2	Stationary bike				
	3	Elliptical bike				
2	4	Stationary bike	3	15	3	50
	5	Treadmill				
	6	Elliptical bike				
3	7	Elliptical bike	2	23	5	60
	8	Stationary bike				
	9	Treadmill				
4	10	Treadmil	2	23	5	60
	11	Treadmil				
	12	Treadmil				
5	13	Treadmill	–	Continuous	-	60
	14	Stationary bike				
	15	Elliptical bike				
6	16	Stationary bike	–	Continuous	–	70
	17	Treadmill				
	18	Elliptical bike				
7	19	Elliptical bike	–	Continuous	–	70
	20	Stationary bike				
	21	Treadmill				
8	22 23	Stationary bike Stationary bike	–	Continuous	–	70
	24	Stationary bike				
9	25	Treadmill	–	Continuous	-	70
	26	Stationary bike				
	27	Elliptical bike				
10	28	Stationary bike	–	Continuous	–	70
	29	Treadmill				
	30	Elliptical bike				
11	31	Elliptical bike	-	Continuous	-	70
	32	Stationary bike				
	33	Treadmill				
12	34	Elliptical bike	-	Continuous	-	70
	35	Elliptical bike				
	36	Elliptical bike				

#### 2.3.3 Concurrent exercise protocol

In exercise sessions of the RES + AER group, the main part combined both exercise protocols. The main part will begin with RT using weights, weight machines, and calisthenics combined with moderate AER on the treadmill, stationary and elliptical bikes. The RT part was divided into two phases: an adaptation period of 3 weeks, followed by 9 weeks of a training-phase; in turn, the moderate AT part included 3 phases. Similarly to the previous two protocols, the RES + AER group intensity will be evaluated by the HRmax and by the Rate of Perceived Exertion Scale progressing to RPE 6–7. Table 3 summarize the main characteristics of the exercise intervention.

**Table 3:** Exercise training plans and progressions-RESISTANCE + AEROBIC (RES + AER) Group.

Week	Session	Anatomic region		N. Exercises	N. Sets	N. Reps	Load percentage (1RM)
1	1	Upper limbs	Back	6	1	15	40
	2	Torso	Upper limbs				
	3	Core	Back				
2	4	Core	Torso	5	2	15–20	50
	5	Upper limbs	Back				
	6	Upper limbs	Upper limbs				
3	7	Torso	Upper limbs	4	2	20	60
	8	Back	Core				
	9	Core	Torso				
4	10	Upper limbs	Upper limbs	4	2	15	60
	11	Upper limbs	Back				
	12	Torso	Upper limbs				
5	13	Back	Core	4	2	15	60
	14	Core	Torso				
	15	Upper limbs	Upper limbs				
6	16	Upper limbs	Back	4	3	6–8	70
	17	Torso	Upper limbs				
	18	Back	Core				
7	19	Core	Torso	4	3	8	70
	20	Upper limbs	Upper limbs				
	21	Upper limbs	Back				
8	22	Torso	Upper limbs	4	3	10	70
	23 24	Back Core	Core Torso				
9	25	Upper limbs	Upper limbs	4	3	12	70
	26	Upper limbs	Back				
	27	Torso	Upper limbs				
10	28	Back	Core	4	3–4	12	70
	29	Core	Torso				
	30	Upper limbs	Upper limbs				
11	31	Upper limbs	Back	4	3–4	12	70
	32	Torso	Upper limbs				
	33	Back	Core				
12	34	Core	Torso	4	3–4	12	70
	35	Upper limbs	Upper limbs				
	36	Upper limbs	Upper limbs				

### 2.4 Outcome measures

#### 2.4.1 Primary aim

The primary outcome is to assess the change in the relative abundance of *Faecalibacterium prausnitzii* and other SCFA-producing bacteria after the intervention (12 weeks). The change in the relative abundance of *Faecalibacterium prausnitzii* was selected as the primary aim given that, to date, there are no studies of exercise effect on GM nor a consensual marker of microbiome health status in these patients (32), and thus, the preliminary data collected in this study, will help us to refine our sample size and study procedures for a full-scale RCT. In addition, it has been shown that patients with sarcopenia have decreased abundance of this bacterial strain- a known SCFA-producing bacteria, with an important role on systemic inflammation, anabolic resistance, insulin sensitivity, and energy production (5, 15). Secondary outcomes are the change in relative abundance of *Faecalibacterium prausnitzii* at close-out (24 weeks), the change in the relative abundance of the genera Lactobacillus and Bifidobacterium after the intervention and at the end of follow-up.

#### 2.4.2 Secondary aims

A set of complementary outcomes will also be assessed to broadly characterize the impact of each exercise intervention, including: body composition, strength, functional performance and overall GM composition. All the methods, instruments and protocols are fully detailed in next sections.

### 2.5 Assessments

Participants will be evaluated in three different moments: (i) baseline; (ii) end of the intervention (12 weeks); (iii) close-out (24 weeks). The study has a total duration of 26 weeks. In each study visit, participants will perform the same physical and functional assessments, body composition analysis, and provide a fasting blood and feces sample. Other confounding variables will also be recorded, including nutritional intake (through a 3-day diary), daily PA levels (through a PA tracker), medication intake. Medication and supplementation along with other associated comorbidities will be assessed using a medical questionnaire and medication history questionnaire. Considering the study design, the following outcomes will also be evaluated: (a) retention and attrition rates; (b) adherence to exercise intervention; (c) safety; (d) intervention effect on dependent outcomes (Table 4).

**Table 4:** Overview of the assessment visits and data collection at each visit.

Study phase	Pre-randomization		Intervention		Follow-up
Visit description	Screening	Baseline	Start	End	CO
Visit number	1	2	3	4	5
Week number	−2	0	2	14	26
Informed consent, review inclusion/exclusion criteria	x				
Demographic information, medical history, medication use	x				
IPAQ questionnaire	x				
Office blood pressure	x	x	x	x	x
Anthropometry measurements	x	x		x	x
Randomization	x				
Body composition (DEXA)		x		x	x
Blood and fecal samples for study assays		x		x	x
Nutritional Intake		x		x	x
Skeletal muscle strength		x		x	x
Physical performance testing		x		x	x
Daily physical activity		x	x	x	x
Assess adverse events			x	x	x
Exercise intervention			x	x	

CO, close-out.

#### 2.5.1 Biospecimen collection—microbiome and metabolomics

At each assessment visit, whole blood and fecal samples will be collected to assess: (a) GM composition and function, i.e., abundance of *Faecalibacterium prausnitzii* and other SCFA-producing bacteria after the intervention; *Lactobacillus* and Bifidobacterium genera after the intervention and at the end of follow-up; relative abundance and diversity at different taxonomic levels such as *Bacillota (Firmicutes)* and *Bacteroidetes* and *Bacteroidetes/Bacillota* ratio; (b) systemic gut-derived metabolites. Venous blood samples will be collected after a fasting period of at least 8 h. These biospecimens will be collected in the morning, between 8 and 10 am in two 5 mL blood tubes. Whole blood will be separated through a centrifugation process for plasma and serum separation, which will be then aliquoted and stored at −80°C for metabolomic assays.

##### 2.5.1.1 Microbiome

Fecal samples will be collected at all assessment visits. Participants will receive a kit composed of: (a) 3-day dietary log record; (b) a fecal collection kit (EasySampler, GP Medical Devices, Holstebro, Denmark); a stool collection tube with DNA stabilizer (Invitek Diagnostics, Berlin, Germany); and (c) instructions to take home about the collection and transportation process. The collection tube has a DNA preservative solution that guarantees the biospecimen integrity for several weeks at room temperature. Samples will be extracted using the PSP Spin Stool DNA Basic Kit from Invitek Diagnostics and GM composition will be analyzed through metabarcoding of fecal samples using high-throughput next generation sequencing (NGS) of amplicon libraries of the V3-V4 region of the 16S rRNA, using the Quick-16S Plus NGS Library Prep Kit (V3-V4, UDI), from Zymo Research (Irvine, CA, United States).

##### 2.5.1.2 Metabolomics

Systemic gut-derived metabolites will be assessed on serum samples using an untargeted liquid chromatography- mass spectrometry (LC–MS) approach, which will quantify circulating metabolites using the Thermo Q-Exactive Orbitrap mass spectrometer with Dionex UHPLC and autosampler. All samples will be analyzed in positive and negative heated electrospray ionization with a Time-of-flight (TOF) survey scan from m/z 50–1,000 collected at 4 Hz throughout each LC–MS run.

#### 2.5.2 Skeletal muscle function

Dominant lower limb knee extension and flexion muscle strength will be assessed through an isokinetic dynamometer, the Biodex System 3 Pro isokinetic dynamometer (System 4 Pro™, Biodex Medical Systems, Shirley, New York, United States) at baseline, end of intervention and close-out. The isokinetic dynamometer is the gold standard method for muscle strength assessment, allowing precise evaluation of force generation within a pre-established range of motion and speed. Test parameters will be defined according to the manufacturer instructions, namely: 90° dynamometer orientation, 0° tilt, 90° seat orientation, 80° seatback tilt and axis of rotation aligned with the lateral femoral condyle determined by inspection and palpation. Participants will be asked to avoid strenuous physical activities in the 48 h prior to testing. Participants will complete a 1 min warm-up consisting of leg extensions and flexions without load while seating in the equipment chair. Afterwards, participants will be fixed to the equipment chair with straps and a passive knee flexion starting from 0° extension will be performed to assess leg weight for gravity correction purposes. Participants will then perform two training sets of 5 reps of isokinetic extension/ flexion at 60°/s, between 0 and 90° range, with a 30s rest interval between sets. After 2 min rest, patients will perform two testing sets of 5 reps with 90s rest interval between sets. Patients will perform the test by exerting maximum possible pressure on the isokinetic arm throughout the entire range of movement. Standardized instructions will be given to encourage maximal muscle performance throughout the test. Peak torque of extension and flexion total work (J) obtained throughout the test in each valid set will be recorded. Maximal muscle strength will be defined as the highest peak torque (Nm) obtained. To avoid sub-maximal performance or outliers, if the peak torque coefficient of variation between the 3 best testing sets exceeds 15%, a new testing set will be performed until a lower than 15% coefficient of variation is obtained between 3 sets. Non-dominant handgrip strength will be assessed using a hand dynamometer (Jamar Plus+ Digital Hand Dynamometer) with participants seated, shoulder adducted in neutral rotation, arm at the side of the body and elbow flexed at 90°. Participants will be encouraged to exert their maximum grip strength. Three repetitions of 3 s will be performed with 30 s interval. The average value of the two repetitions with the lowest coefficient of variation will be considered.

#### 2.5.3 Anthropometry and body composition

Body weight (BW) and height will be measured using a precision scale coupled to a stadiometer (Seca 285). The Body Mass Index (BMI) will be calculated using the BMI = weight/height^2^ equation. Waist and hip circumferences will be measured with a measuring tape (Seca 203) according to WHO guidelines (33), and then waist-to-hip and waist-to-height ratios will be calculated. Body composition and percentage body mass will be assessed by whole-body DXA (DXA; Hologic Explorer QDR) according to the manufacturer recommendations and using standard protocols for positioning and analysis of the DXA scans. The same DXA equipment will be used throughout the experimental period. All scans will be performed and analyzed by the same trained researcher. Phantoms will be scanned daily, and coefficients of variation will be analyzed during the experimental period to ensure reliability. In every assessment (baseline, end of the intervention, and close-out) each participant will perform a whole-body scan for total and regional body composition. The outcome variables obtained with DXA are total and regional lean mass (kg, %), total and regional fat mass (kg, %), absolute appendicular skeletal muscle mass, relative appendicular skeletal muscle mass per weight, height and BMI. All participants will be assessed between 8 and 10 am following a > 8 h fasting period while wearing only light cloths. For whole-body composition assessment, participants will lie supine with arms alongside, legs in extension and slight internal rotation with feet attached by a Velcro strap. Anthropometric measurements and body composition will be assessed using appropriate procedures and gold standard methods.

#### 2.5.4 Nutritional intake

All participants will be instructed to record their total food and drink intake during the three consecutive days prior to the day of fecal collection using a food diary. At each of the three scheduled assessment visits (baseline, end of the intervention and close-out), all participants will receive a small brochure, with pre-printed forms, in which they will record the information about the time, type and amount of food and beverages consumed. Together with the delivery of the diary, participants will receive instructions with an explanation and practical examples on how they should record this information. After the 3 days of recording, participants will return the diary booklet (together with the fecal collection sample). The first A5 size booklet, will be delivered in the subsequent experimental periods to help standardize the nutritional intake. This will aid in the analysis of other parameters, since differences in fiber, protein and total energy intake between groups may significantly interfere with the interpretation of the primary and secondary outcomes. After all nutritional data has been collected, food intake will be encoded, and a comprehensive nutrient analysis will be performed with the Food Processor Plus software (ESHA Research).

#### 2.5.5 Physical performance

The physical performance of the participants will be evaluated at baseline, end of the intervention and at close-out using gait speed, the 30-s chair rise, the Short Physical Performance Battery (SPPB) and the 400 meter walk test as recommended by the EWGSOP guidelines (27). To assess gait speed, we will use the 4-m usual walk test with speed time measured with a stopwatch. A cut-off speed ≤0.8 m/s is an indicator of severe sarcopenia while 1.0 m/s is a sign of physical function limitations. The 30-s chair rise test counts how many times a participant can rise and sit in the chair over a 30-s interval and evaluates lower limb strength and endurance. The SPPB is a composite test battery that includes assessment of gait speed, a balance test, and a chair stand test. Each test is scored 4 points until a maximum score of 12 points. In contrast a score of ≤8 points indicates poor physical performance. Lastly, in the 400-meter walk test, participants are asked to complete 20 laps of 20 m, each lap as fast as possible, and are allowed up to two rest stops during the test. This test evaluates walking ability and endurance. These tests are expressed in a continuous quantitative scale that allows the assessment of longitudinal changes gradually over time (improvements or decline). Participants will be evaluated between 8 and 10 a.m. and will be instructed to avoid vigorous exercise in the 2 h prior to testing to minimize intraday variability, temperature effects, and biological rhythms. In addition, participants will be asked to wear comfortable clothes and appropriate walking shoes and to continue their usual medication. The recommended reasons to stop the physical performance testing included: chest pain, intolerable dyspnea, leg cramps, staggering, diaphoresis, and pale or ashen appearance. Participants will be evaluated in the same testing order: (1) gait speed (m/s); (2) the SPPB (score); (3) 30-s chair rise (count); and (4) the 400-m walk test (s).

#### 2.5.6 Additional assessment procedures

Per the study design, we will also evaluate: (a) retention and attrition rates; (b) adherence to exercise intervention; (c) safety. The success of recruitment and retention rates will be measured by the number of participants recruited, the number of drop-outs and losses to follow-up throughout the trial. To consider a dropout, the participant must miss at least two consecutive training weeks (*n* = 6 training sessions) without any explanation nor successful contact from the study team. Adherence to the exercise intervention will be carefully documented by study staff and will be measured by the number of sessions attended. Safety will be measured by the number and/or seriousness of adverse events attributable to the intervention and will be monitored and recorded by study staff throughout the trial.

The clinical and medication history will be assessed by the General Health and Physical Activity Questionnaire, an adapted version (34). This questionnaire will also collect the smoking habits and participants’ demographic information. Office BP will be assessed on the left arm after 5 min rest, in the seated position with a digital sphygmomanometer (Efficia, CM150, Philips). Physical inactivity will be assessed by the IPAQ – Portuguese version. Participants with ≤150 min/week of moderate PA or without regular exercise training habits will be classified as physically inactive (35).

### 2.6 Statistical analysis

Descriptive and exploratory analyses will be performed, and normality will be assessed using the Kolmogorov–Smirnov or Shapiro–Wilk tests, and if necessary, the data will be transformed for normalization. The differences between the baseline groups will be explored using one-way analysis of variance (ANOVA) or Kruskal-Wallis tests for continuous data and chi-square tests for categorical data.

The intention-to-treat principle will be used for the primary outcome analysis. The main analyses will employ random coefficient linear mixed models for normally distributed endpoints or generalized mixed models for non-normally distributed endpoints. Post-hoc analysis with Bonferroni correction will assess differences between treatment factors. Cohen’s d will be used to indicate the effect size, and significance will be set at *p* < 0.05.

Fecal samples will undergo taxonomic analysis, grouped into amplicon sequence variants (ASVs), and summarized at different taxonomic levels. These data will be imported in R for use with the Phyloseq package. Several indices of alpha and beta diversity will be evaluated (i.e., Simpson Index, Shannon-Wiener Index, Bray–Curtis dissimilarity, Unweighted and weighted Uni-Frac for beta-diversity). Principal Coordinate Analysis (PCoA) will be performed to visualize the dissimilarity matrix between all samples and a PERMANOVA on exercise groups will be performed. Analysis of Compositions of Microbiomes (ANCOM) with bias correction will be used to test for differential abundance of individual ASVs at several levels to compare the three exercise groups. We will use the Benjamin– Hochberg corrected significance level of 0.05 to minimize false discovery rate (FDR). For the metabolomics analysis, samples will be normalized by the total ion current and means centered using Pareto scaling. Univariate analysis of variance will be performed on data from both ion modes to identify variations in peak areas between groups at baseline and after the 12-weeks intervention. Partial least squares discriminant analysis (PLS-DA) will be used to detect the most critical discriminant metabolites in each comparison across groups consistent with published guidelines for metabolomics trials (36). Microsoft packages (e.g., Excell), SPSS (version 27) and R (version 4.2.) software will be used to manage and analyze the different data.

The study primary null hypothesis are: (a) there are no differences in *Faecalibacterium prausnitzii* and other SCFA-producing bacteria after the intervention between groups; (b) there are no differences in *Faecalibacterium prausnitzii* and the other SCFA-producing bacteria after the intervention between the RES + AER group versus the AER group; (c) there are no differences in *Faecalibacterium prausnitzii* and other SCFA-producing bacteria after the intervention between the RES + AER group vs. the RES group. We hypothesize that combining AT and RT will have a greater impact on the GM composition, specifically in increasing the relative abundance of *Faecalibacterium prausnitzii* and other SCFA producing bacterial strains, an increase in skeletal muscle mass and strength, as well as enhanced physical performance measures in the concurrent exercise training group.

## 3 Discussion

To date, there are no studies on the effects of exercise on GM nor a consensual marker of gut microbiome health status in older adults with sarcopenia. This paucity of data and the scarcity of evidence limits, not only the clinical application of the optimal exercise prescription regimen in clinical context but also by exercise specialists among these high-risk patients. The existence of the gut-muscle axis adds a novel dimension to our understanding of the pathophysiological mechanisms of sarcopenia and raises the attractive possibility that by manipulating the GM composition and function, we could mediate changes in beneficial bacterial taxa associated to skeletal muscle health (e.g., *Faecalibacterium praunitzii*) and prevent sarcopenia and its adverse health outcomes (37).

The increasing prevalence of sarcopenia demands effective treatment interventions to alleviate the personal, social, and economic burden associated with this condition. It has been estimated that a reduction of sarcopenia by 10% in the US healthcare system would save up to $ 1.1 billion per year. Thus, our study will contribute to this specific goal by evaluating the impact of different exercise training regimens on a new emerging regulator of skeletal muscle mass and function (i.e., gut microbiome) which has been linked to functional impairment. Our study will expand the current knowledge in this area and will provide precise information to prescribe the optimal treatment plan in these patients, stimulating an active intervention to preserve people’s autonomy. In addition, this project will provide the preliminary data to further investigate other underlying unknown mechanistic links that mediates different exercise training modalities and contribute to a consecutive line of research aimed at unraveling the role of gut microbiome and its multiple interaction mechanisms in exercise training in patients with sarcopenia.

Aging decreases dominant species such as *Bacteroidaceae*, *Lachnospiraceae*, and R*uminococcaceae* and increases subdominant species, particularly, opportunistic bacteria (38). Several studies correlate changes in the diversity of the GM with poor physical performance in older adults, and an increase in the representation of *Faecalibacterium prausnitzii, Roseburia inulinivorans*, and *Alistipes shahii* in the feces of non-sarcopenic people compared to sarcopenic stools (5, 39). The abundance of *Faecalibacterium prausnitzii*—known for its important role in the health of the gut microbiota (i.e., in insulin sensitivity, anabolic balance, and inflammation), appears in some studies with a representation up to 5 times lower in individuals with sarcopenia (5, 18). *Bifidobacteriaceae, Dialister, Pyramidobacter* and *Eggerthella* and the depletion of *Slackia* and *Eubacterium* were also increased in older adults with physical frailty and sarcopenia, when compared to non-frail individuals (40). Therefore, therapeutic strategies developed to modulate GM composition may have clinical meaningful implications in age-related muscle loss and physical function decline.

While research into sarcopenia treatments (nutraceuticals to pharmacological strategies) surged over the past decade, only physical exercise, especially RT (27), alone or in combination with AT, was proven effective in enhancing health outcomes and functionality in older adults (41). Clinical studies also show that combining exercise with dietary supplements (protein or fiber) effectively fights sarcopenia (42). Whether these changes in patients with sarcopenia are paralleled by modifications of GM remains to be explored.

A recent review summarizing data from interventional and observational studies on older adults (where sarcopenia was more prevalent) indicated that exercise and physical activity can have a beneficial impact on the GM composition (19) but due to methodological and sampling disparities, it was not possible to reach a consensus on which taxa was most responsive/influenced by exercise. Further, growing evidence shows that different exercise training modalities (type, frequency, duration, and intensity) may elicit different GM composition phenotypes and skeletal muscle health outcomes (18). Propionate and butyrate, both gut-derived SCFA metabolites, can act as adenosine monophosphate-activated protein kinase (AMPK) activators and stimulate peroxisome proliferator-activated receptor-gamma coactivator 1 alpha isoform (PGC-1α) and insulin/Insulin-like Growth Factor 1 (IGF-1) signaling, promoting muscle biogenesis, whereas its decrease has physiological consequences like reduction in the mitochondrial fatty acids’ oxidation, increased intramuscular fatty acid deposition and insulin resistance (1, 11). Thus, given that different exercise training regimens differently impact the body, we can hypothesize that each regimen promotes distinct alterations on GM composition. RES may enhance bacterial strains associated to muscle strength while AER may promote the increase on the relative abundance of taxa associated to aerobic capacity and SCFA production whereas the combination of both forms of exercise (resistance + aerobic) may lead to an added effect. Furthermore, with this study we will be able to shed light on the (ir)reversibility of the preliminary results, evaluate the adequacy of our primary outcome and analyze the effect of our intervention. The findings collect on the washout period will be important to investigate GM resilience (i.e., the ability or process of the GM to withstand exercise benefits and/or manage everyday challenges) and specifically, explore whether these changes induced by exercise are sustained over time or not in the three different exercise regimens.

*In summary*, with this study we expect to create a consecutive line of research to fill a current literature gap and explore the impact of three different exercise regimens on GM composition and gut-derived metabolites in older adults with sarcopenia. These findings may help clinical guidance and enhance exercise prescription in this high-risk population. For instance, if confirmed that RES + AER are the optimal mode of exercise training to modulate GM composition and improve skeletal muscle health, clinicians and exercise specialists may prioritize it in patients with sarcopenia with certain GM composition phenotypes. Lastly, our study will contribute with an evidence-based approach, to a deeper understanding on the optimal exercise mode to elicit gut and skeletal muscle health benefits. Therefore, our study could have clinical meaningful implications and impact patient’s quality of life and lifespan.

## Data Availability

The original contributions presented in the study are included in the article/supplementary material, further inquiries can be directed to the corresponding author.

## Footnotes

^1^ www.randomizer.org

## Abbreviations

1RM: one-repetition maximum
AER: Moderate Aerobic Exercise
AMPK: Adenosine Monophosphate-Activated Protein Kinase
ANCOM: Analysis of Compositions of Microbiomes
ASVs: Amplicon sequence variants
AT: Aerobic Training
BMI: Body Mass Index
BW: Body Weight
CERT: Consensus on Exercise Reporting Template
CIAFEL: Research Center in Physical Activity, Leisure and Health
CV: Cardiovascular
DBP: Diastolic Blood Pressure
EWGSOP: European Working Group on Sarcopenia in Older People
FDR: False discovery rate
GM: Human Gut Microbiota
HR: Heart Rate
HR_max_: Maximum Heart Rate
IGF**-1**: Insulin-like Growth Factor 1
IPAQ: International Physical Activity Questionnaire
IPATIMUP: Institute of Molecular Pathology and Immunology of the University of Porto
LC–MS: Liquid chromatography- mass spectrometry
NGS: Next generation sequencing
PA: Physical Activity
PCoA: Principle Coordinate Analysis
PLS**-DA**: Partial least squares discriminant analysis
RCT: Randomized Control Trial
RES: Resistance Exercise
RES + AER: Concurrent Exercise Training
RPE scale: Rate of Perceived Exertion Scale
RT: Resistance Training
SBP: Systolic Blood Pressure
SCFA: Short-Chain Fatty Acids
SPPB: Short Physical Performance Battery
TOF: Time-of-flight
UA: University of Aveiro
UMIB: Unit Multidisciplinary Research in Biomedicine
